# Elevated levels of PAI-1 precede the occurrence of type 2 diabetes mellitus

**DOI:** 10.1186/s13098-025-01629-4

**Published:** 2025-02-18

**Authors:** Jenny Hernestål-Boman, Tina Öhman, Jan-Håkan Jansson, Marcus M. Lind, Olov Rolandsson, Ingvar A. Bergdahl, Lars Johansson

**Affiliations:** 1 Department of Laboratory Medicine, University Hospital of Umeå, Umeå, Sweden; 2 Department of Public Health and Clinical Medicine, Umeå University, Umeå, Sweden; 3 Department of Medicine, Skellefteå Lasarett, Skellefteå, S-931 86 Sweden

**Keywords:** Type 2 diabetes, Plasminogen activator inhibitor-1, Population study, Västerbotten intervention programme

## Abstract

**Aims:**

Plasminogen activator inhibitor-1 (PAI-1) is the main inhibitor of the fibrinolytic system and is mainly secreted from adipose tissue. It is associated with cardiovascular disease and has also been considered a possible early risk marker for type 2 diabetes. Here, we present the results of a large prospective study investigating PAI-1 levels in relation to incident type 2 diabetes mellitus.

**Methods:**

We conducted a prospective incident case-referent study within the Västerbotten Intervention Programme (VIP). Data on cardiovascular risk factors, fasting plasma glucose (FPG) and 2-hour plasma glucose (2-hPG) were collected at baseline health examination 1990–2005. Blood samples were collected and stored for future analyses. Participants were followed and 484 cases developed type 2 diabetes. Referents without type 2 diabetes were matched for sex, age, and year of participation, *n* = 484. Baseline plasma samples were analysed for PAI-1. Subgroup analysis was performed for 201 cases and 201 matched referents with normal baseline glucose levels (FPG < 6.1 and 2hPG < 8.9 mmol/L).

**Results:**

Elevated baseline levels of PAI-1 were associated with incident type 2 diabetes after adjustments for BMI, family history of diabetes, smoking status, hypertension, FPG and 2hPG (PAI-1; OR = 1.87, 95% CI: 1.06–3.29). A similar result was shown in the subgroup analysis with 201 participants who had normal glucose levels at time of the health examination (PAI-1; OR = 2.68, 95% CI: 1.03–6.95).

**Conclusions:**

Elevated PAI-1 levels in non-diabetic persons precede the manifestation of type 2 diabetes and can be detected before an elevation of FPG or 2-hPG is observed.

**Supplementary Information:**

The online version contains supplementary material available at 10.1186/s13098-025-01629-4.

## Introduction

Established risk factors for the development of type 2 diabetes include overweight, smoking, physical inactivity, high blood lipids, high blood pressure, and a family history of type 2 diabetes [1]. Plasminogen activator inhibitor-1 (PAI-1) is the main inhibitor of the fibrinolytic system and is mainly secreted from adipose tissue. PAI-1 is a marker for increased risk of cardiovascular disease. In a previous study, we included 157 diabetes-free participants that later developed type 2 diabetes and found that elevated levels of PAI-1 at baseline preceded the manifestation of type 2 diabetes [2]. In addition, several studies have reported significantly elevated PAI-1 levels in patients with type 2 diabetes compared to controls [3–7]. A systematic review of the epidemiological literature, supports a link between PAI-1 and risk for type 2 diabetes [8]. The conclusion was that elevated PAI-1 levels appear to be associated with incident type 2 diabetes independently of established diabetes risk factors, and that there is a need for investigation of the role of PAI-1 on diabetes risk within cohorts with normal and increased glucose levels at baseline.

The present, larger, study aims to investigate if levels of PAI-1 were associated with incident type 2 diabetes, and secondly, to explore if elevated PAI-1 levels precede the elevation of fasting plasma glucose (FPG) and 2-hour plasma glucose (2-hPG).

## Methods

### Study population

**Fig. 1:**
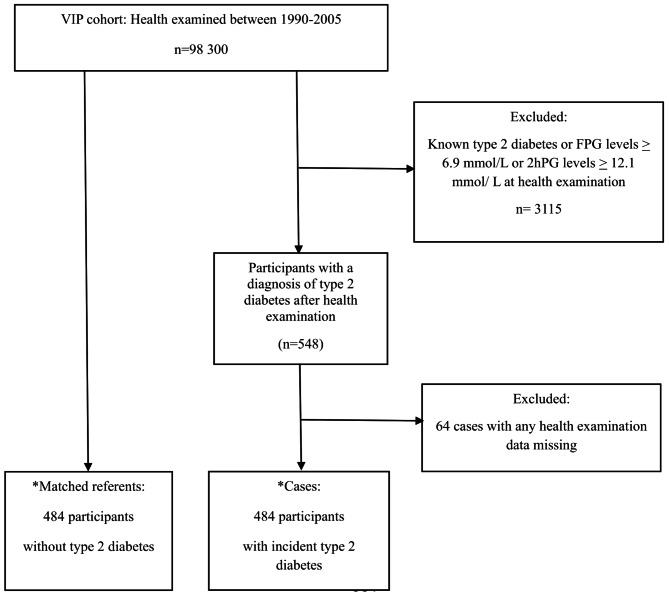
Flow chart showing the selection of cases and referents in the VIP cohort *All included cases and referents have a complete set of data Referents are matched for age, sex, and year of health examination

This is a nested case-referent study within the Västerbotten Intervention Programme (VIP) cohort [9]. Inhabitants in the Västerbotten County in the north of Sweden were invited to their local primary-care centre when they were 40, 50, or 60 years old to participate in a health survey. Health examination included measurement of height, weight, blood pressure, fasting blood glucose, oral glucose tolerance test, and a questionnaire including questions about smoking habits, physical activity and family history of diabetes. The examination was followed by a motivational health promotion dialogue with a trained nurse. Participants were asked to donate a blood sample for future research. The present study included 98 300 subjects who were health examined in VIP between 1990 and 2005, see Fig. 1. The participation rate was high; the total study population represented 70% of the eligible population [10]. A study on selection bias showed that there were small differences between participants and non-participants [11].

Participants were followed between 1990 and 2005 and 484 cases diagnosed with incident type 2 diabetes were included via a local diabetes register (DiabNorth) according to WHO criteria [12]. One referent, without type 2 diabetes according to the registry in 2005, was randomly selected for each case, matched for age, sex, and year of health examination. Complete data sets regarding PAI-1, BMI, fasting and 2-hour glucose levels, total cholesterol, blood pressure, physical activity, smoking and family history of diabetes were found in 484 cases with diagnosed type 2 diabetes during follow-up and 484 matched referents. Participants with known type 2 diabetes or fasting plasma glucose (FPG) levels above 6.9 mmol/L or 2-hour plasma glucose (2hPG) levels above 12.1 mmol/L at baseline were excluded.

### Measurements

Participants were instructed to fast from midnight until time of blood sampling. Venous blood samples were drawn with a minimum of stasis in a sitting position into evacuated tubes containing 1/100 volume of 0.5% EDTA. Each tube was centrifuged at 1500 x *g*; the plasma was immediately frozen at -20 °C and stored at -80 °C until analysis. Oral glucose tolerance tests were performed with a 75-g glucose load according to WHO standards. FPG was measured in venous plasma in a fasting state and in capillary plasma 2 h after glucose intake on a Reflotron bench-top analyser (Boeringer Mannheim GmbH, Mannheim, Germany). Total cholesterol was analysed on thawed frozen samples and performed via routine methods at the Department of Clinical Chemistry at Umeå University Hospital. ELISA reagent kits for PAI-1 were purchased from TrioLab (Gothenburg, Sweden). The inter-assay coefficient of variation for PAI-1 analysis was 4.4%. Samples from cases and their matched referent were analysed together in random order. All reagent kits had identical batch number. Measurements were made in 2016 by laboratory staff unaware of each subject’s disease status.

BMI was calculated as measured weight in kilograms divided by the square of the height in metres (kg/m2). Smoking was categorized into two groups (ever or never smoking). Participants were considered to have a family history of diabetes if they reported having a parent or sibling with type 2 diabetes. Physical activity was categorized into two levels based on the questionnaire: inactive or active. Hypertension was defined as systolic blood pressure of 140 mmHg or more and/or diastolic blood pressure of 90 mmHg or more and/or reported use of antihypertensive medication during the period of 14 days prior to the health examination.

### Statistical methods

Data are presented as proportion (%), mean and standard deviation (SD). Significance testing was carried out using independent sample’s t-test for continuous variables and Chi-Squared test for categorical variables.

Univariable conditional logistic regression was used to calculate age- and sex-matched Odds ratios (OR) comparing the risk of incident type 2 diabetes in cases and referents. Multivariable conditional regression analysis was performed to estimate the effects of potential confounders when controlling for factors with *p* < 0.2 in the univariable analyses. ORs with 95% Confidence Intervals (CI) were calculated per increment of 1 SD for continuous variables. Only case-referent pairs with complete data sets were included.

To investigate whether PAI-1 increases prior to increased levels of plasma glucose we used the subgroup of case-referent pairs where both individuals had normal glucose levels (FPG < 6.1 mmol/L and 2hPG < 8.9 mmol/L) at baseline. Multivariable analysis was again performed for potential confounders as described above.

The Statistical Package for Social Science (SPSS^®^ version 27.0) was used for statistical analysis. A *p*-value < 0.05 (two-sided) was considered statistically significant.

### Ethical considerations

This study protocol was approved by the Research Ethics Committee of Umeå University. All participants provided written informed consent.

## Results

During a mean follow-up time of 7.6 years, 484 cases developed type 2 diabetes. The mean age at health examination was 49 years and 48% were women, see Table 1. Cases had higher PAI-1 levels compared to referents as well as higher BMI, systolic blood pressure, FPG and 2hPG. More cases than referents were ever smokers, had hypertension and a family history of type 2 diabetes. PAI-1 levels were correlated to age, BMI, FPG, 2hPG, systolic blood pressure and total cholesterol levels among referents, with the strongest correlation with BMI (rs = 0.46) (Supplemental Table 1).

**Table 1:** Baseline characteristics of 484 cases with incident type 2 diabetes mellitus and their matched referents

		Cases *n* = 484	Referents *n* = 484		*p*-value
Age, years		48.8 ± 7.3	48.8 ± 7.3		matched
Sex, female n (%)		232 (47.9)	232 (47.9)		matched
Time to T2DM (years)		7.6 ± 3.1	n.a.		
BMI, kg/m2		29.0 ± 4.5	25.3 ± 3.4		< 0.001
Family history of T2DM, n (%)		167 (34.5)	79 (16.3)		< 0.001
Smoking, n (%)					0.012
	Never	172 (35.5)	210 (43.4)		
	Ever	312 (64.5)	274 (56.6)		
Physical activity, n (%)					0.33
	Active	208 (43.0)	223 (46.1)		
	Inactive	276 (57.0)	261 (53.9)		
Systolic blood pressure, mmHg		137 ± 17.3	127 ± 16.5		< 0.001
Hypertension, n (%)		259 (53.5)	129 (26.7)		< 0.001
Total cholesterol, mmol/L		5.9 ± 1.2	5.8 ± 1.1		0.39
PAI-1, ng/mL		66.2 (30.5)	45.7 (24.4)		< 0.001
FPG, mmol/L		5.8 ± 0.7	5.4 ± 0.6		< 0.001
2-hPG, mmol/L		7.9 ± 1.9	6.5 ± 1.4		< 0.001

Values are mean ± SD or numbers and proportions

T2DM: type 2 diabetes mellitus

PAI-1: plasminogen activator inhibitor-1

FPG: fasting plasma glucose

2-hPG: 2-hour plasma glucose

Elevated levels of PAI-1 were independently associated with incident type 2 diabetes (OR = 1.87, 95% CI: 1.06–3.29) after multivariable analysis including adjustments for BMI, family history of diabetes, smoking status, hypertension, FPG and 2hPG (Table 2).

**Table 2:** Conditional logistic regression for 484 cases with incident type 2 diabetes compared to their 484 matched referents

		Crude OR (95% CI)		Adjusted model† OR (95% CI)
BMI		3.66 (2.14–6.24)		2.49 (1.38–4.49)
Family history of T2DM		2.75 (1.22–6.18)		4.03 (1.04–15.7)
Smoking				
	Never	1		
	Ever	2.00 (1.00–4.00)		1.48 (0.43–5.12)
Physical activity				
	Active	1		
	Inactive	0.92 (0.53–1.61)		
Hypertension		4.00 (1.84–8.68)		1.79 (0.51–6.34)
Total cholesterol		1.04 (0.76–1.42)		
PAI-1		2.49 (1.63–3.79)		1.87 (1.06–3.29)
FPG		1.61 (1.19–2.19)		1.24 (0.73–2.09)
2-hPG		2.43 (1.62–3.64)		1.76 (0.97–3.18)

Odds ratio (OR) and 95% CI per 1 SD increment. Only varaibles with crude *p* < 0.2 were included in the adjusted model

T2DM: type 2 diabetes mellitus

PAI-1: plasminogen activator inhibitor-1

FPG: fasting plasma glucose

2-hPG: 2-hour plasma glucose

**†** Adjusted model: Adjusted for body mass index, family history of T2DM, smoking, hypertension, PAI-1, FPG and 2hPG

Likewise, in the subgroup of 201 cases and matched referents with normal glucose levels (FPG < 6.1 mmol/L and 2hPG < 8.9 mmol/L) at baseline, PAI-1 concentrations were independently associated with incident type 2 diabetes in the multivariable model (PAI-1; OR = 2.68, 95% CI: 1.03–6.95), see Table 3.

**Table 3:** Conditional logistic regression for 201 cases and their 201 matched controls with normal levels of fasting plasma glucose (FPG) and 2-hour plasma glucose (2-hPG) and risk for incident type 2 diabetes mellitus

		Crude OR (95% CI)		Adjusted model† OR (95% CI)
BMI		2.91 (1.63–5.21)		1.94 (0.94-4.00)
Family history of T2DM		3.60 (1.34–9.70)		6.44 (1.37–30.3)
Smoking				
	Never	1		
	Ever	1.71 (0.68–4.35)		
Physical activity				
	Active	1		
	Inactive	0.79 (0.36–1.73)		
Hypertension		4.00 (1.34–11.97)		1.81 (0.16–4.04)
Total cholesterol		0.92 (0.62–1.36)		
PAI-1		2.61 (1.77–7.36)		2.68 (1.03–6.95)
FPG		0.84 (0.53–1.33)		
2-hPG		2.46 (1.31–4.62)		2.30 (0.83–6.36)

Odds ratio (OR) and 95% CI per 1 SD increment. Only varaibles with crude *p* < 0.2 were included in the adjusted model

T2DM: type 2 diabetes mellitus

PAI-1: plasminogen activator inhibitor-1

FPG: fasting plasma glucose

2-hPG: 2-hour plasma glucose

† Adjusted model: Adjusted for body mass index, family history of T2DM, hypertension, PAI-1 and 2hPG

## Discussion

In the present study, elevated levels of PAI-1 were associated with incident type 2 diabetes, independently of established diabetes risk factors. This was true also for participants with normal blood glucose levels at health examination. These associations were independent of family history of type 2 diabetes, glucose levels, BMI and other risk factors for diabetes.

We suggest that elevated PAI-1 is an early link in the chain of events leading to type 2 diabetes. Excess visceral adipose tissue has been shown to increase PAI-1 secretion [13, 14], perhaps as a result of the chronic inflammation associated with obesity [15].

Several studies have shown the association of type 2 diabetes and increased levels of PAI-1 [8]. A meta-analysis by Yarmolinsky et al., found a link between PAI-1 and T2DM, independent of established diabetes risk factor. As the association was moderate and there was heterogeneity across the studies further prospective studies were suggested. No individual prospective study in the meta-analysis included more than 182 cases. In the present study we included 484 cases and the association between PAI-1 levels and incident type 2 diabetes remained significant after adjustments for several potential confounders. Furthermore, in participants with normal glucose levels at the health examination only PAI-1 and family history of diabetes were shown to be significantly associated with incident type 2 diabetes.

Alessi et al. have shown that adipose tissue produces PAI-1 [16], but also that insulin stimulates the synthesis of PAI-1 [17]. PAI-1 itself has been shown to inhibit insulin signaling by competing with αvβ-3 integrin for vicronectin binding [18] and by disturbing receptor substrate − 1 activity and expression [19]. Troglitazone treatment in insulin-resistant lean or obese type 2 diabetes patients can lower PAI-1 concentrations and is associated with enhanced fibrinolytic activity linked to lower plasma insulin levels and improved glycemic control [20]. Also, after being fed the same high fat diet, obesity and insulin resistance were completely prevented in mice lacking PAI-1, compared to wild-type mice [21].

In the promoter region of the PAI-1 gene there is a well-known 4G/5G polymorphism [22]. Obese women with the 5G/5G genotype has been shown to have significantly lower levels of plasma PAI-1 than the 4G/4G group [23]. However, in a multivariate analysis including insulin resistance, polymorphisms had a minor contribution to the PAI-1 variability (3% in women and no significant results in men), which indicate that PAI-1 levels depend more on metabolic changes than polymorphisms [24].

### Strengths and limitations

This study has a prospective case-referent design with diabetes-free men and women aged 40 to 60 years when participating in an extensive baseline health examination. Participants who developed type 2 diabetes were examined on average 7.6 years before the manifest diagnosis. Diabetic patients were defined according to WHO criteria. Storage time has been shown to have a negligible impact on laboratory PAI-1 measurements of frozen samples [25].

The study was conducted within a well-defined intervention programme, the VIP, where recommended lifestyle changes, such as weight loss and smoking cessation, may have influenced the incidence of type 2 diabetes [26]. Most participants were of Caucasian origin limiting the possibility of generalizing the results to populations of other ethnicities. The results were based on one single baseline blood sample per participant, which may decrease the estimations of the observed associations.

## Conclusions

Elevated PAI-1 levels in non-diabetic persons precede the manifestation of type 2 diabetes and can be detected before an elevation of FPG or 2-hPG is observed.

## Electronic supplementary material

Below is the link to the electronic supplementary material.

Supplementary Material 1

Supplementary Material 2

## Data Availability

No datasets were generated or analysed during the current study.

## Abbreviations

2hPG: 2-hour plasma glucose after oral glucose tolerance test
BMI: Body mass index
CI: Confidence interval
FPG: Fasting plasma glucose
OGTT: Oral glucose tolerance test
PAI-1: Plasminogen activator inhibitor-1
SD: Standard deviation
type 2 diabetes: Type 2 diabetes mellitus
VIP: Västerbotten Intervention Programme
