# Supplementary material for: Elevated levels of PAI-1 precede the occurrence of type 2 diabetes mellitus
Source: Diabetol Metab Syndr. 2025 Feb 18;17:61. doi: 10.1186/s13098-025-01629-4 (PMC11834294; doi:10.1186/s13098-025-01629-4)
Supplement: Supplementary file 1 — Supplementary Material 1 [file 13098_2025_1629_MOESM1_ESM.docx]

**Supplemental table: Baseline characteristics of 201 cases with incident type 2 diabetes mellitus and their matched referents**

|  | |  | |  | |
| --- | --- | --- | --- | --- | --- |
|  | | **Cases**  **n=201** | **Referents**  **n=201** | | **p-value** |
| Age, years | | 47.0±7.1 | 47.0±7.1 | | matched |
| Sex, female n (%) | | 88 (43.8) | 88 (43.8) | | matched |
| Time to T2DM, years | | 8.5 (2.9) | n.a. | |  |
| BMI, kg/m2 | | 28.7 ± 4.2 | 25.0 ± 3.4 | | < 0.001 |
| Family history of T2DM, n (%) | | 73 (36.3) | 30 (14.9) | | < 0.001 |
| Smoking, n (%) | |  |  | | 0.21 |
|  | Never | 67 (33.3) | 79 (39.3) | |  |
|  | Ever | 134 (66.7) | 122 (60.7) | |  |
| Physical activity, n (%) | |  |  | | 0.23 |
|  | Active | 78 (38.8) | 90 (48.8) | |  |
|  | Inactive | 123 (61.2) | 111 (55.2) | |  |
| Systolic blood pressure, mmHg | | 134 ± 16.6 | 126 ± 16.4 | | < 0.001 |
| Hypertension, n (%) | | 95 (47.3) | 42 (20.4) | | < 0.001 |
| Total cholesterol, mmol/L | | 5.9 ± 1.2 | 5.8 ± 1.1 | | 0.39 |
| PAI-1, ng/mL | | 67.9 (32.0) | 43.1 (25.5) | | < 0.001 |
| FPG, mmol/L | | 5.3 ± 0.6 | 5.3 ± 0.4 | | 0.25 |
| 2-hPG, mmol/L | | 6.9 ± 1.2 | 6.2 ± 1.1 | | < 0.001 |

Values are mean ± SD or numbers and proportions

T2DM: type 2 diabetes mellitus

PAI-1: plasminogen activator inhibitor-1

FPG: fasting plasma glucose

2-hPG: 2-hour plasma glucose
