# Supplementary material for: Elevated levels of PAI-1 precede the occurrence of type 2 diabetes mellitus
Source: Diabetol Metab Syndr. 2025 Feb 18;17:61. doi: 10.1186/s13098-025-01629-4 (PMC11834294; doi:10.1186/s13098-025-01629-4)
Supplement: Supplementary file 2 — Supplementary Material 2 [file 13098_2025_1629_MOESM2_ESM.docx]

**Supplementary table 1: Spearman correlations of 968 cases and referents**

|  | Age | PAI-1 | BMI | FPG | 2-hPG | SBP | TC |
| --- | --- | --- | --- | --- | --- | --- | --- |
| Age | 1 |  |  |  |  |  |  |
| PAI-1 | 0.09* | 1 |  |  |  |  |  |
| BMI | 0.001 | 0.46** | 1 |  |  |  |  |
| FPG | 0.13* | 0.26** | 0.27** | 1 |  |  |  |
| 2-hPG | 0.20** | 0.23** | 0.25** | 0.29** | 1 |  |  |
| SBP | 0.26** | 0.25** | 0.33** | 0.10 | 0.26** | 1 |  |
| TC | 0.18** | 0.14** | 0.06 | 0.08 | 0.03 | 0.12** | 1 |

2-hPG: 2-hour plasma glucose

FPG: fasting plasma glucose

PAI-1: plasminogen activator inhibitor-1

SBT: systolic blood pressure

TC: total cholesterol

* Correlation is significant at the 0.05 level (2-tailed)

** Correlation is significant at the 0.01 level (2-tailed)
